# Markers of human endometrial hypoxia can be detected *in vivo* and *ex vivo* during physiological menstruation

**DOI:** 10.1093/humrep/deaa379

**Published:** 2021-01-26

**Authors:** J J Reavey, C Walker, M Nicol, A A Murray, H O D Critchley, L E Kershaw, J A Maybin

**Affiliations:** 1 MRC Centre for Reproductive Health, The Queen’s Medical Research Institute, Edinburgh, UK; 2 Edinburgh Imaging, The Queen’s Medical Research Institute, Edinburgh, UK; 3 Centre for Inflammation Research, The Queen’s Medical Research Institute, Edinburgh, UK

**Keywords:** endometrium, menstrual, menses, progesterone, hypoxic, perfusion, MRI, menorrhagia, gynaecology

## Abstract

**STUDY QUESTION:**

Can markers of human endometrial hypoxia be detected at menstruation *in vivo*?

**SUMMARY ANSWER:**

Our *in vivo* data support the presence of hypoxia in menstrual endometrium of women during physiological menstruation.

**WHAT IS KNOWN ALREADY:**

Current evidence from animal models and human *in vitro* studies suggests endometrial hypoxia is present at menstruation and drives endometrial repair post menses. However, detection of human endometrial hypoxia *in vivo* remains elusive.

**STUDY DESIGN, SIZE, DURATION:**

We performed a prospective case study of 16 women with normal menstrual bleeding.

**PARTICIPANTS/MATERIALS, SETTING, METHODS:**

Reproductively aged female participants with a regular menstrual cycle underwent objective measurement of their menstrual blood loss using the alkaline haematin method to confirm a loss of <80 ml per cycle. Exclusion criteria were exogenous hormone use, an intrauterine device, endometriosis or fibroids >3 cm. Participants attended for two MRI scans; during days 1–3 of menstruation and the early/mid-secretory phase of their cycle. The MRI protocol included dynamic contrast-enhanced MRI and T2* quantification. At each visit, an endometrial sample was also collected and hypoxia-regulated repair factor mRNA levels (*ADM*, *VEGFA*, *CXCR4*) were quantified by RT-qPCR.

**MAIN RESULTS AND THE ROLE OF CHANCE:**

Women had reduced T2* during menstrual scans versus non-menstrual scans (*P* = 0.005), consistent with menstrual hypoxia. Plasma flow (F_p_) was increased at menstruation compared to the non-menstrual phase (*P* = 0.0005). Laboratory findings revealed increased *ADM, VEGF-A* and *CXCR4* at menstruation on examination of paired endometrial biopsies from the menstrual and non-menstrual phase (*P* = 0.008; *P* = 0.03; *P* = 0.009). There was a significant correlation between T2* and these *ex vivo* hypoxic markers (*P* < 0.05).

**LIMITATIONS, REASONS FOR CAUTION:**

This study examined the *in vivo* detection of endometrial hypoxic markers at specific timepoints in the menstrual cycle in women with a menstrual blood loss <80 ml/cycle and without significant uterine structural abnormalities. Further research is required to determine the presence of endometrial hypoxia in those experiencing abnormal uterine bleeding with and without fibroids/adenomyosis.

**WIDER IMPLICATIONS OF THE FINDINGS:**

Heavy menstrual bleeding (HMB) is a common, debilitating condition. Understanding menstrual physiology may improve therapeutics. To our knowledge, this is the first *in vivo* data supporting the presence of menstrual hypoxia in the endometrium of women with normal menstrual bleeding. If aberrant in those with HMB, these non-invasive tests may aid diagnosis and facilitate personalized treatments for HMB.

**STUDY FUNDING/COMPETING INTEREST(S):**

This work was funded by Wellbeing of Women grant RG1820, Wellcome Trust Fellowship 209589/Z/17/Z and undertaken in the MRC Centre for Reproductive Health, funded by grants G1002033 and MR/N022556/1. H.O.D.C. has clinical research support for laboratory consumables and staff from Bayer AG and provides consultancy advice (but with no personal remuneration) for Bayer AG, PregLem SA, Gedeon Richter, Vifor Pharma UK Ltd, AbbVie Inc; Myovant Sciences GmbH. H.O.D.C. receives royalties from UpToDate for articles on abnormal uterine bleeding.

**TRIAL REGISTRATION NUMBER:**

N/A.

## Introduction

Heavy menstrual bleeding (HMB) affects 20–30% of pre-menopausal women causing decreases in physical, psychological, economic and social health (RCOG, 2011). Medical treatments are available but are often discontinued due to lack of efficacy or side effects. A national 4-year audit of HMB in the UK reported that 43% of women received surgery in the year following first attendance at hospital (RCOG, 2011). Surgery usually ends fertility and introduces the risk of organ damage, haemorrhage and infection. Delineation of the physiology of menstrual initiation and cessation is required to identify effective diagnostic and personalized therapeutic strategies for this debilitating symptom.

Progesterone levels decline in the late secretory phase of the menstrual cycle due to corpus luteal regression. This causes an influx of inflammatory mediators to the local endometrial environment, including potent vasoconstrictors, e.g. prostaglandin-F_2α_ and endothelin-1 (Smith *et al.*, 1981; Marsh *et al.*, 1997). This ‘injury’ leads to bleeding and shedding of the functional endometrial layer during menstruation. The endometrium then undergoes re-epithelialization and stromal expansion in an efficient ‘repair’ phase to maintain function (i.e. receptivity) in the subsequent cycle (Critchley *et al.*, 2020).

Hypoxia arises when the metabolic oxygen demand of a tissue exceeds its supply and is usually defined as a partial pressure of less than 10 mmHg (Hockel and Vaupel, 2001). The presence and role of hypoxia during human endometrial breakdown and repair remains controversial. Its presence during menstruation was first suggested 80 years ago. Endometrial explants transplanted into the anterior chamber of the eye in the rhesus monkey revealed that progesterone withdrawal was followed by intense vasoconstriction, tissue necrosis and bleeding (Markee, 1940). Endometrial hypoxia has been confirmed during menstruation in the mouse model of simulated menses using pimonidazole, a marker of oxygen levels <10 mmHg (Fan *et al.*, 2008; Cousins *et al.*, 2016; Maybin *et al.*, 2018). However, xenograft studies in mice failed to detect hypoxia in human endometrial explants during endometrial breakdown or repair (Coudyzer *et al.*, 2013).

Our human *ex vivo* studies have revealed that hypoxia-inducible factor (HIF)-1α and its downstream targets can be detected in human endometrial biopsies, exclusively during the peri-menstrual phase, with a distinct reduction when bleeding has ended (Critchley *et al.*, 2006; Maybin *et al.*, 2018). HIF-1α is rapidly degraded in normoxia and is the master regulator of the hypoxic response, hence its presence is consistent with a local tissue hypoxia in the endometrium during menstruation.

*In vitro*, progesterone withdrawal and hypoxia are necessary for induction of putative repair factors in the human endometrial explants, e.g. vascular endothelial growth factor (VEGF). The hypoxic induction of these factors was dependent upon HIF-1α (Maybin *et al.*, 2011a,c). We hypothesize that endometrial hypoxia is present in women during physiological menstruation to drive the repair process and limit menstrual blood loss.

Accurate determination of human endometrial perfusion and hypoxia *in vivo* has not been possible to date. Non-invasive magnetic resonance techniques have been used to investigate markers of tissue hypoxia at other tissue sites (Punwani *et al.*, 1998; Prasad and Epstein, 1999; Jordan *et al.*, 2004; Nielsen *et al.*, 2004; dos Santos *et al.*, 2007). T2* is a characteristic tissue relaxation time. It depends on inhomogeneities in the main magnetic field produced by the scanner as well as inhomogeneities induced by the presence of other nearby molecules such as deoxyhaemoglobin, a marker of tissue oxygenation (Ogawa *et al.*, 1990). The presence of paramagnetic deoxyhaemoglobin within red blood cells increases their magnetic susceptibility, creating local magnetic field gradients around the cells (Thulborn *et al.*, 1982) which results in a reduction in T2* (Jezzard *et al.*, 2003). This technique has been widely applied in both pre-clinical and clinical studies to evaluate tissue oxygenation status in organs including the kidney (Prasad and Epstein, 1999; dos Santos *et al.*, 2007), brain (Punwani *et al.*, 1998) and muscle (Jordan *et al.*, 2004).

Dynamic contrast-enhanced MRI (DCE-MRI) enables quantitative estimation of tissue perfusion parameters. It analyses the temporal enhancement of a tissue after a paramagnetic (gadolinium-based) contrast agent is injected intravascularly. These images allow quantification of parameters such as vascular plasma volume (v_p_), plasma flow (F_p_), extraction fraction (E) and permeability surface area product (PS). This technique has been used to investigate tissue perfusion in the breast (Kuhl *et al.*, 1999), prostate (Preziosi *et al.*, 2003), heart (Nielsen *et al.*, 2004) and liver (Patel *et al.*, 2010). We propose that these magnetic resonance techniques could be applied to investigate endometrial perfusion and hypoxia non-invasively.

The objective of this study was to determine if endometrial hypoxia can be detected *in vivo* using MRI. Herein, we describe the use of T2* quantification and DCE-MRI to determine the presence of markers of hypoxia in the endometrium of women with normal menstrual bleeding.

## Materials and methods

### Ethical approval

A favourable ethical opinion for these studies was obtained from the West of Scotland Research Ethics Committee (REC 15/WS/0212).

### Recruitment

Women of reproductive age (18–55 years) with regular menstrual cycles (21–35 days) were invited to participate. Women with exogenous hormone or intrauterine device use in the two months prior to recruitment and those with known endometriosis or fibroids >3 cm were excluded. Written informed consent was obtained from 31 women (Supplementary Fig. S1). Three women withdrew from the study and four became ineligible (one woman became pregnant, one woman commenced fertility treatment and two women entered the peri-menopause with onset of menstrual cycle irregularity). Therefore, 24 participants underwent objective measurement of their menstrual blood loss using the alkaline haematin method (Hallberg and Nilsson, 1964). Sixteen women had an objective blood loss of <80 ml and were included in the study. These participants were invited to attend for two MRI scans, one in the menstrual phase of their cycle (Days 1–3) and the other timed for the early/mid-secretory phase. Further confirmatory evidence of correct cycle stage was provided by (i) serum blood sample for measurement of circulating oestradiol and progesterone concentrations and (ii) endometrial histological appearance (Noyes *et al.*, 1950) in women who also had an endometrial biopsy collected. Participant characteristics and hormone levels are detailed in Supplementary Table SI.

### MRI assessment

Each participant was imaged supine at 3T (Siemens Magnetom Verio or Skyra^fit^, Siemens Healthineers, Erlangen, Germany), once during the menstrual phase (Days 1–3) and once during the early/mid-secretory phase of their cycle. Prior to imaging, 20 mg hyoscine butylbromide was administered intravenously in order to minimize bowel peristalsis and improve image quality. Images were acquired using the 32-channel (Verio) or 60-channel body array (Skyra^fit^). Fourteen women completed two scans, one woman did not attend a second scan and one woman had a non-menstrual sample classified as ‘disordered proliferative’ and was excluded from analysis. Of the 14 women who completed two scans, two women were unable to complete the full protocol (n = 1 had T2* quantification only and n = 1 underwent DCE-MRI only).

### T2*

**Figure 1.**
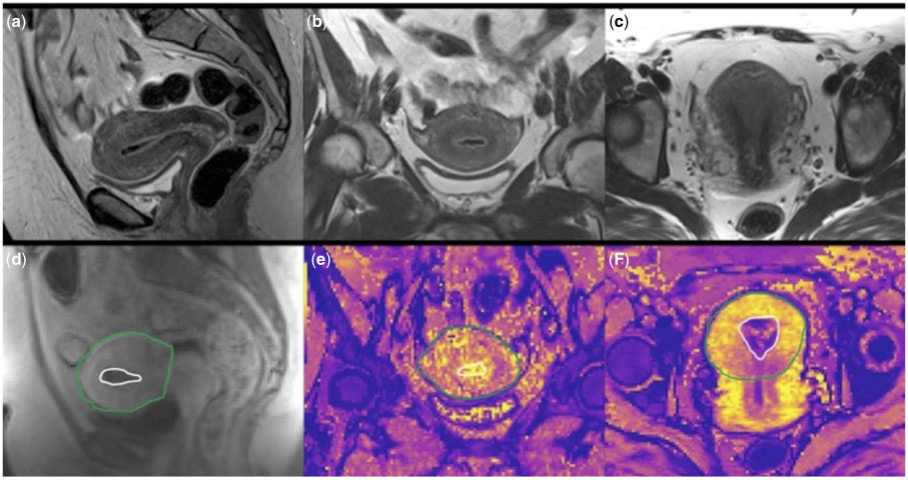
**Example images and regions of interest.** Top row: T2-weighted (T2w) structural images in the sagittal (**a**), coronal (**b**) and axial planes (**c**), slice locations matching bottom row images for easy reference during outlining. Bottom row: Dynamic contrast-enhanced image (**d**) showing outlined endometrium (white) and uterus (green). T2* maps in the coronal (**e**) and axial (**f**) planes with endometrium and uterus outlined.

For quantification of T2*, a single slice multiecho gradient echo in the axial and coronal planes (Siemens MyoMaps) was used (sequence parameters are detailed in Supplementary Table SII). Regions of interest (ROI) for the endometrium and uterus (excluding the cervix) were delineated using OsiriX. The axial and coronal T2* images were examined with reference to structural T2-weighted (T2w) sagittal, coronal and axial images (TSE, Turbo Spin Echo) (Fig. 1a–c) to help clearly delineate the ROI (Fig. 1e and f). T2* mean and standard deviation were calculated for each ROI. When ROIs for both planes in the T2* images were able to be obtained, the T2* value from the plane which had a lower standard deviation was selected. In some planes for the T2* images, the ROI could not be clearly delineated due to poor resolution. If this occurred then the same planes were used across the two scans for the participant when possible (i.e. coronal, coronal or axial, axial).

### DCE-MRI

The perfusion imaging protocol included IR-TrueFISP (inversion recovery true fast imaging with steady-state precession) for T1 measurement and a sagittal dynamic 3D FLASH (fast low angle shot) which was acquired during injection of 0.1 mg/kg Gadovist followed by 20 ml saline chaser at 2 ml/s. The sequence parameters used are in Supplementary Table SII. T1 maps were calculated using in-house software (Python 3.6) by fitting a standard inversion recovery curve to the IR-TrueFISP data. This was then used to convert signal intensity to contrast agent concentration (CA) in the dynamic images. The two-compartment uptake model (de Bazelaire *et al.*, 2005; Ingrisch and Sourbron, 2013) was fitted to concentration versus time curves on a voxel-by-voxel basis, using arterial input functions derived at each visit from the common iliac arteries. In this model, the CA is assumed to be distributed between two compartments: the plasma (vascular) space (v_p_) and the extravascular extracellular space (EES). The CA is delivered to the plasma space via the arteries at a rate depending on the F_p_. A fraction of the CA diffuses into the EES at a rate depending on the permeability and surface area of the capillary walls (PS). In the two-compartment uptake model, the assumption is made that the backflow of CA from the EES to the plasma space is negligible. This model was fitted for F_p_, v_p_ and E which is the fraction of CA extracted from the plasma into the EES in the first pass. E is related to PS via the equation E = PS/(PS + F_p_) (Sourbron and Buckley, 2011).

OsiriX was used to draw ROIs for the endometrium and uterus (excluding the cervix) on the high resolution sagittal T2w images. These ROIs were then transferred to the lower resolution dynamic series images with adjustments if required (Fig. 1d). Mean values and standard deviations of F_p_, E and v_p_ for the uterus and endometrium were extracted from calculated parameter maps using the pyOsiriX plugin (Blackledge *et al.*, 2016).

### Tissue collection

Endometrial biopsies were collected with an endometrial suction curette (Pipelle, Laboratoire CCD, Paris, France) and immediately divided and placed in (i) RNA later (Ambion (Europe) Ltd., Warrington, UK) prior to storage at −80°C and (ii) 4% (v/v) formaldehyde (10% neutral buffered formalin) prior to paraffin embedding. Tissue was dated according to three criteria: (i) histological appearance (Noyes *et al.*, 1950) assessed by a consultant pathologist (ii) serum levels of oestradiol and progesterone at time of biopsy and (iii) date of the participant’s last menstrual period. Of the 16 women who participated, five women wished for a single endometrial biopsy only, two women declined an endometrial biopsy and in one woman it was not possible to obtain an endometrial sample. Therefore, eight women had paired endometrial samples in the menstrual and non-menstrual phase (proliferative n = 1; early/mid-secretory n = 7) (Supplementary Fig. S1).

### Real-time quantitative reverse transcription PCR

Total RNA from human endometrium samples was extracted using the RNeasy Mini Kit (Qiagen Ltd, Sussex, UK) according to manufacturer’s instructions. RNA samples (100 ng) were reverse transcribed using iScript cDNA synthesis kit (Bio-Rad Laboratories Ltd, UK) alongside control samples. Specific primers were designed using the universal probe library assay design centre and checked with BLAST (Supplementary Table SIII). All primers were validated before use to ensure efficiency was 90–100%. Samples were analysed in triplicate using ABI QuantStudio system. The comparative 2-ΔΔCt method was used to analyse genes of interest. Normalization to the geometric mean of the reference genes (*SDHA* and *ATP5B*) was performed before comparison to a positive calibrator sample (liver cDNA). These reference genes were determined using the geNorm assay and shown to be stable across stage of menstrual cycle.

### Statistical analysis

Statistical analysis was performed using GraphPad Prism Software (GraphPad Prism Software, Inc., San Diego, CA, USA). Paired *t*-tests (normally distributed data) or the Wilcoxon signed-rank test (non-parametric data) was used to analyse differences in MRI quantification and PCR values between menstrual and non-menstrual cycle stages. Correlation between MRI and laboratory detection of hypoxic markers was determined using the Spearman’s rank correlation test. A value of *P < *0.05 was considered statistically significant.

## Results

### Endometrial T2* was significantly lower at menstruation versus non-menstrual timepoints, consistent with the presence of endometrial hypoxia at menses

**Figure 2.**
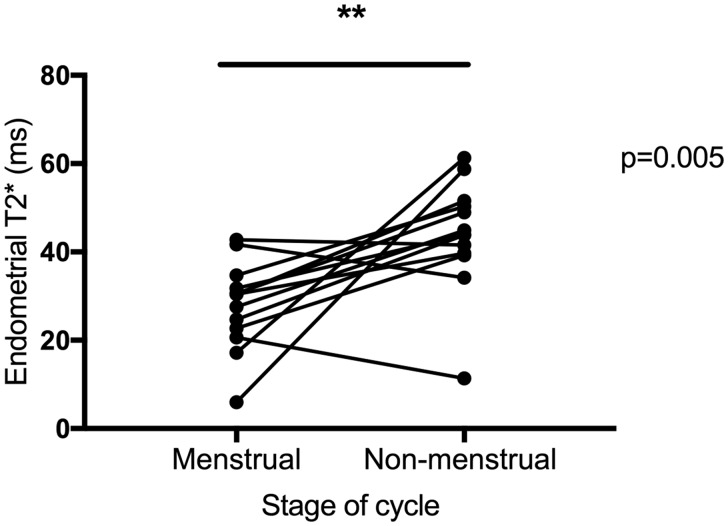
Endometrial T2* was significantly lower at menstruation than during the non-menstrual phase, consistent with menstrual endometrial hypoxia. n = 13 women scanned on two visits. **P < 0.01.

Endometrial T2* was quantified in 13 participants with confirmed normal menstrual bleeding (<80 ml) during the menstrual phase (Days 1–3) and a non-menstrual timepoint (n = 12 early/mid-secretory, n = 1 proliferative). Endometrial T2* was significantly lower in the menstrual phase compared to the non-menstrual phase (*P* = 0.005) consistent with the presence of endometrial hypoxia at menses (Fig. 2).

### Endometrial plasma flow was significantly higher at menstruation versus non-menstrual timepoints

**Figure 3.**
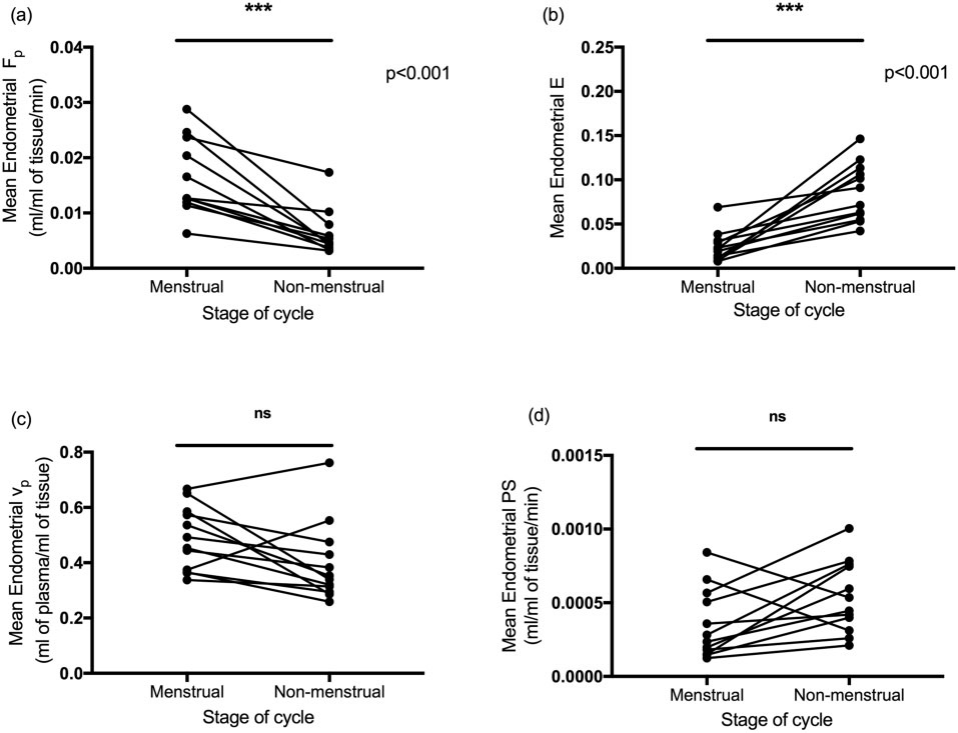
**Endometrial plasma flow (F_p_) was significantly higher at menstruation than during the non-menstrual phase.** (**a**) Endometrial F_p_ at menstruation and the non-menstrual phase. (**b**) Endometrial extraction fraction (E) at menstruation and the non-menstrual phase. (**c**) Endometrial plasma volume (v_p_) at menstruation and the non-menstrual phase. (**d**) Endometrial permeability surface area product (PS) at menstruation and the non-menstrual phase. n = 13 women scanned on two visits; ns, not significant; ***P < 0.001.

DCE-MRI analysis was completed on 12 paired visits (as one participant was excluded from final analysis due to non-physiological F_p_ values). Endometrial F_p_ was significantly higher in the menstrual phase compared to the non-menstrual phase (*P* < 0.001) (Fig. 3a). Endometrial E was significantly lower at menstruation compared to the non-menstrual phase (*P* < 0.001) (Fig. 3b). There was no difference in endometrial v_p_ between these two cycle phases (*P* = 0.05) (Fig. 3c).

To further interpret the significant decrease in endometrial E at menstruation, the PS was calculated and compared. No difference in endometrial PS between the two cycle phases was seen (*P* = 0.06) (Fig. 3d). Therefore, the difference in E is driven by the change in flow (Fp) rather than a change in vessel permeability between different cycle stages.

### *Ex vivo* hypoxic markers were significantly higher in human menstrual endometrium compared with endometrium when not menstruating

**Figure 4.**
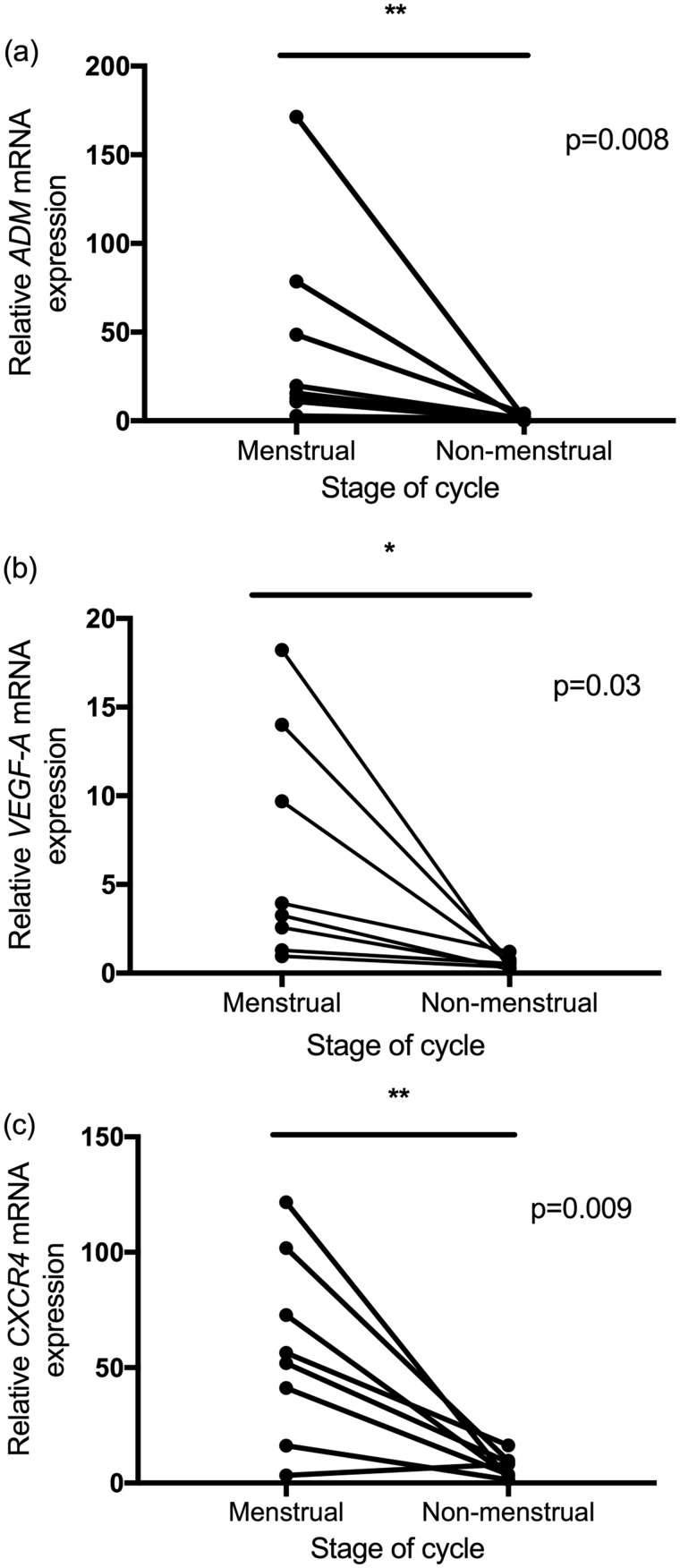
***Ex vivo* hypoxia markers were significantly higher in menstrual versus non-menstrual endometrium.** (**a**) Endometrial *ADM* mRNA levels at menstruation and the non-menstrual phase. (**b**) Endometrial *VEGF-A* mRNA levels at menstruation and the non-menstrual phase. (**c**) Endometrial *CXCR4* mRNA levels at menstruation and the non-menstrual phase. n = 8 paired biopsies; *P < 0.05; **P < 0.01.

RNA was extracted from whole endometrial tissue and HIF-1 regulated genes quantified using RT-qPCR. Analysis was performed on eight paired menstrual and non-menstrual samples. There were significantly higher levels of *ADM*, *VEGF-A* and *CXCR4* in menstrual compared to non-menstrual endometrium (*P* < 0.01, *P* < 0.05, *P* < 0.01, respectively) (Fig. 4a–c).

### The significant negative correlation between T2* values and RT-qPCR concentrations of endometrial hypoxia-regulated genes highlight the potential of MRI as a tool to detect endometrial hypoxia *in vivo*

**Figure 5.**
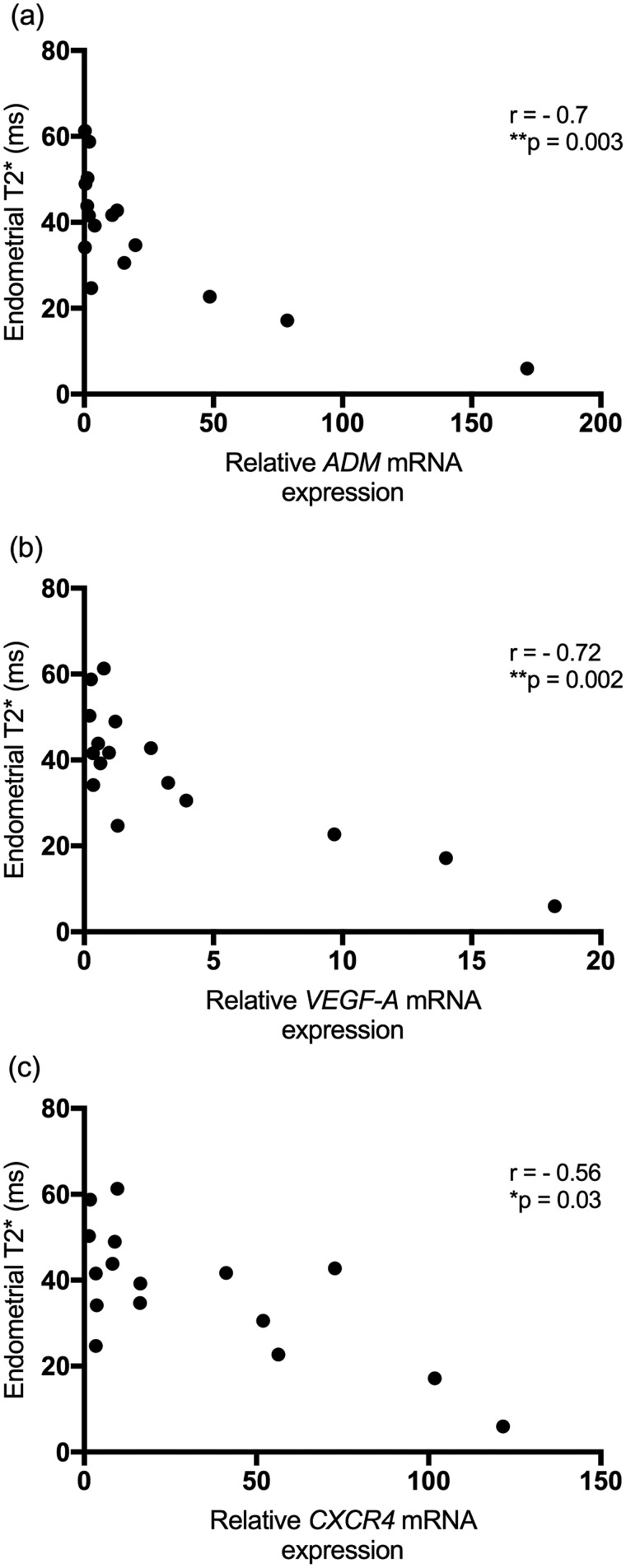
Endometrial T2* is significantly correlated with endometrial concentrations of hypoxia-regulated genes (a: ADM; b: *VEGF-A* and c: *CXCR4*) indicating a potential role in non-invasive detection of endometrial hypoxia. n = 8 paired biopsies/T2*; *P < 0.05; **P < 0.01.

To validate T2* quantification as a non-invasive technique for assessing endometrial hypoxia, Spearman’s rank correlation was performed between T2* and *ADM*/*VEGF-A* and *CXCR4* mRNA levels in menstrual and non-menstrual endometrium from women with normal menstrual bleeding. A significant negative correlation was seen between T2* and the concentrations of these hypoxia-regulated genes (*P* < 0.05) (Fig. 5a–c).

## Discussion

Here, we report that women with normal menstrual bleeding have significantly altered MRI parameters during menstruation compared to those detected during non-menstrual phases of the cycle. T2* values were significantly decreased during menstruation, consistent with increased deoxyhaemoglobin and tissue hypoxia at menses. Endometrial plasma flow was significantly increased during menstruation, whereas extraction fraction was decreased and plasma volume was not different to non-menstrual values. *Ex vivo* detection of downstream targets of HIF-1 were significantly increased in menstrual versus non-menstrual endometrial tissue, consistent with menstrual endometrial hypoxia.

To our knowledge, these data are the first to report endometrial T2* at two different time points of the menstrual cycle. T2* quantification has previously been reported in healthy uterine myometrium across the cycle (Kido *et al.*, 2007; Imaoka *et al.*, 2012). The junctional zone of the myometrium, located adjacent to the endometrium, was found to have a lower T2* value than the outer myometrium (Kido *et al.*, 2007; Imaoka *et al.*, 2012). This may be secondary to physiological uterine contractions and peristalsis within the subendometrial junctional zone (Nakai *et al.*, 2003), resulting in increased deoxyhaemoglobin. Furthermore, consistent with our findings in the endometrium, the T2* value of both the junctional zone and the outer myometrium were lower in the menstrual phase compared to values throughout the rest of the cycle (Kido *et al.*, 2007), which may be secondary to reduced uterine myometrial perfusion during menstruation.

A number of other paramagnetic substances, e.g. ferritin and haemosiderin, can cause a change in the local magnetic field and may affect T2*. The presence of ferritin in human endometrial samples has been investigated previously by immunohistochemistry (Tsionou *et al.*, 1991). Ferritin was not detected in benign endometrial samples but was present in endometrial hyperplasia and carcinoma samples (Tsionou *et al.*, 1991). Menstrual endometrial fragments have been used to induce endometriosis in mice (Van Langendonckt *et al.*, 2004). This study found that, prior to injection, the majority of menstrual endometrial fragments were negative for Prussian blue (a stain that detects ferritin). Therefore, it is unlikely that ferritin contributes to the changes in T2* seen in this study. Haemosiderin, an iron storage complex, has been detected in the endometrium of women using intrauterine levonorgestrel devices (Phillips *et al.*, 2003) but does not occur in normal, cycling endometrium as the tissue sloughs during menstruation (Murdock *et al.*, 2019). Haemosiderin deposition is a feature of chronic bleeding rather than the acute bleeding of normal menstrual endometrial breakdown. Finally, brain imaging also uses T2* to evaluate intracranial haematomas. Following acute haemorrhage, oxyhaemoglobin is progressively deoxygenated. At 12–48 h following acute bleeding, deoxyhaemoglobin is predominant within the haematoma (Kidwell and Wintermark, 2008). In contrast to haematoma formation, menstrual bleeding is a dynamic process with tissue shedding and then passing through the cervix. There is evidence that levels of plasminogen activator increase pre-menstrually (Casslen and Astedt, 1981) to prevent clot organization and enable tissue fragments to pass through the cervical os. Therefore, we believe it is unlikely that the T2* changes demonstrated were due to the presence of old blood within the uterine cavity at menstruation. Hence, we propose our finding of reduced T2* at menstruation is consistent with endometrial hypoxia.

Our DCE-MRI findings were unexpected as we hypothesized that vasoconstriction of endometrial vessels at menstruation would reduce plasma flow and volume. Support for vasoconstriction following progesterone withdrawal comes from a number of human studies. Administration of mifepristone (an anti-progestin) in the secretory phase of the cycle resulted in subsequent increased immunoreactivity for COX-2 (Hapangama *et al.*, 2002), the enzyme responsible for the synthesis of the vasoconstrictor prostaglandin-F_2α_. A further study administering mifepristone to women in the secretory phase found this resulted in decreased lumen area of the endometrial capillaries, consistent with reduced blood flow (Johannisson *et al.*, 1989). In the classical experiments by Markee (1940), vasoconstriction of the Rhesus macaque spiral arterioles was also observed following progesterone withdrawal. However, importantly this was seen to occur prior to menstruation. Therefore, we propose that endometrial spiral arteriole vasoconstriction may occur transiently in the late secretory phase and this constriction has resolved by days 1–3 of the cycle. Hence, instead of capturing spiral arteriole vasoconstriction, we may have detected the subsequent perfusion changes in the endometrium during our imaging window. Reactive hyperaemia is the temporary increase in tissue blood flow that occurs to restore tissue oxygen levels following a period of oxygen deprivation (Bliss, 1998). The increase in plasma flow that we detected at menstruation may reflect reactive hyperaemia due to the physiological mechanism of ‘hypoxic vasodilation’ (Roy and Brown, 1880; Heistad and Wheeler, 1970; Rowell and Blackmon, 1989). This prompt vascular response increases perfusion of blood to oxygen-deprived tissues and has been well described in cerebral vessels (Milledge and Sorensen, 1972; Wilson *et al.*, 2011; Willie *et al.*, 2012), the coronary circulation (Herrmann and Feigl, 1992; Martinez *et al.*, 2005) and skeletal muscle vascular beds (Black and Roddie, 1958; Halliwill, 2003). Future DCE-MRI studies should focus on the late secretory phase to determine if endometrial perfusion is decreased prior to the onset of active bleeding.

We demonstrate a significant increase in hypoxia-regulated factors *ADM*, *VEGF-A* and *CXCR4* at menstruation. A number of previous studies investigating putative endometrial repair factors with known HIF-response elements (e.g*. CXCR4*, *CXCL12*, *ADM*) across the menstrual cycle are limited by their lack of inclusion of menstrual endometrium (Shifren *et al.*, 1996; Laoag-Fernandez *et al.*, 2000; Laird *et al.*, 2011). Published gene expression datasets do not enable comparison of hypoxia-regulated genes between menstrual and secretory endometrium in those with normal menstrual blood loss (Ponnampalam *et al.*, 2004; Girling *et al.*, 2017; Wang *et al.*, 2020). However, consistent with our findings herein, there is existing evidence that *ADM*, *VEGF and CXCR4* mRNA levels are significantly up-regulated in the human endometrium at menstruation (Maybin *et al.*, 2011a,c; 2018) and that menstrual endometrial stromal cells from the functional layer may contribute to the repair process (Gaide Chevronnay *et al.*, 2009). To our knowledge, our data are the first to demonstrate increased menstrual hypoxia-regulated genes in the endometrium using the more robust methodology of paired samples from the same woman at two stages of the menstrual cycle.

A significant negative correlation between T2* and *ADM*/*VEGF-A*/*CXCR4* was shown, providing further support for T2* quantification as a non-invasive technique to detect markers of endometrial hypoxia. T2* quantification has been widely used to investigate tissue oxygenation in the kidney (Prasad and Epstein, 1999; dos Santos *et al.*, 2007), brain (Ogawa *et al.*, 1990; Punwani *et al.*, 1998), muscle (Jordan *et al.*, 2004) and prostate (Hoskin *et al.*, 2007; Chopra *et al.*, 2009). A close correlation has previously been demonstrated between T2* and the hypoxic marker pimonidazole (McPhail and Robinson, 2010) along with oxygen levels measured using a fibre-optic probe (dos Santos *et al.*, 2007), needle electrode (Chopra *et al.*, 2009) and near-infrared spectroscopy (Punwani *et al.*, 1998). We believe the data presented herein are the first to support T2* quantification for detection of hypoxia in the human endometrium. This highlights the potential of this technique to assess the hypoxic response in the human endometrium at menstruation non-invasively and may present a novel diagnostic test for aberrant hypoxia at menstruation.

Although physiological oxygen tensions vary across tissues, the majority of human end organ tissues are not hypoxic (<10 mmHg). Reported oxygen tensions in normal human tissue range from the lower end in muscle (29 mmHg) and brain (35 mmHg) to higher oxygen tensions in liver (41 mmHg) and intestinal tissue (58 mmHg) (Koh and Powis, 2012). The actual oxygen tension at the human endometrial surface during menstruation is unknown. To date, studies have investigated this only in non-menstrual endometrium. One study used a fibre-optic microsensor to measure oxygen tension at the endometrial surface in women just prior to intrauterine insemination (on cycle day 11-18) and found an average oxygen tension of 18.9 mmHg (Ottosen *et al.*, 2006). This was in keeping with an earlier study using an oxygen microelectrode that recorded an average endometrial oxygen tension of 15 mmHg across the proliferative, ovulatory and secretory phase (Yedwab *et al.*, 1976). These results from non-menstrual endometrium suggest that with spiral arteriole vasoconstriction, occurring just prior to menstruation, it is possible that the oxygen tension may fall below 10 mmHg in menstrual endometrium.

An aberrant hypoxic response at menstruation may contribute to HMB of endometrial origin, i.e. those with no anatomical abnormalities or systemic disorders and regular menstrual cycles (Munro *et al.*, 2018). There is evidence that women with HMB have reduced peri-menstrual endometrial expression of vasoconstrictors, such as endothelin-1 and prostaglandins (Maybin and Critchley, 2011). As the radius of a vessel is the major determinant of resistance to flow, decreased constriction of endometrial vessels at menstruation will contribute significantly to HMB (Maybin *et al.*, 2011b). Lack of vasoconstriction during menstruation will also prevent peri-menstrual hypoxia in the endometrium of women with HMB. Our previous studies have detected decreased HIF-1α protein and its downstream targets in the menstrual endometrium of women with HMB (Maybin *et al.*, 2018). Furthermore, using our mouse model of simulated menstruation, we have novel *in vivo* data demonstrating that pharmacological inhibition of HIF-1α during menses resulted in delayed re-epithelialization and stromal restoration and its stabilization leads to accelerated repair (Maybin *et al.*, 2018). Therefore, future studies using these MRI techniques require inclusion of women with HMB to determine if they can be used to non-invasively detect aberrant endometrial hypoxia *in vivo*.

In summary, our results have identified altered MRI parameters in the menstrual endometrium of women with normal menstrual blood loss that are consistent with endometrial hypoxia at menses. These techniques have the potential to diagnose aberrant physiological hypoxia at menstruation to facilitate precise treatment strategies for those with problematic menstrual bleeding.

## Data availability

The data underlying this article will be shared on reasonable request to the corresponding author.

## Supplementary Material

deaa379_Supplementary_FigureS1

deaa379_Supplementary_TableSI

deaa379_Supplementary_TableS2

deaa379_Supplementary_TableS3
