# Supplementary material for: Markers of human endometrial hypoxia can be detected in vivo and ex vivo during physiological menstruation
Source: Hum Reprod. 2021 Jan 26;36(4):941–50. doi: 10.1093/humrep/deaa379 (PMC7970728; doi:10.1093/humrep/deaa379)
Supplement: deaa379_Supplementary_FigureS1 [file deaa379_supplementary_figures1.pdf]

Human Reproduction, pp. 1–1, 2021
doi:10.1093/humrep/deaa379

human reproduction

SUPPLEMENTARY DATA

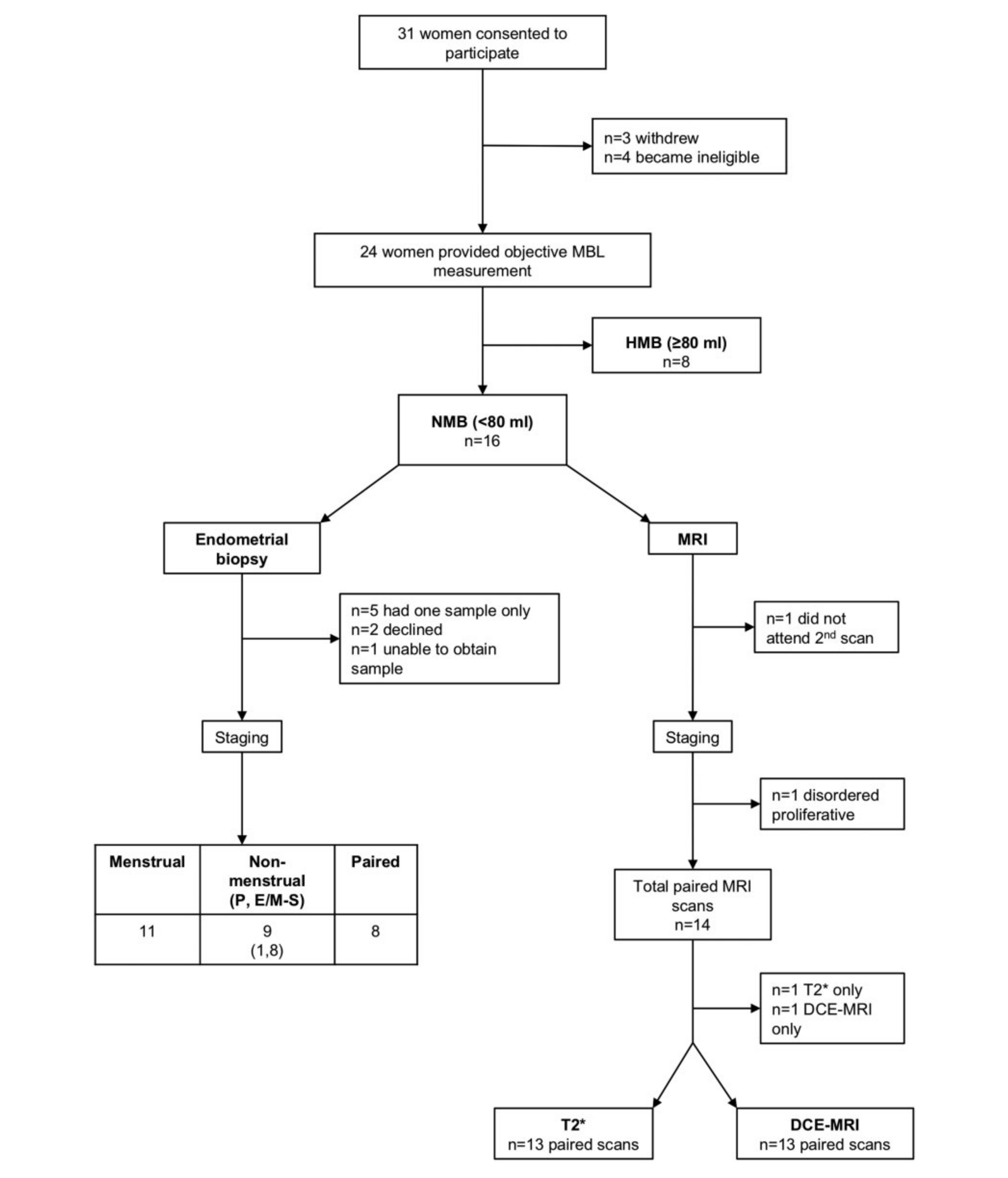

Supplementary Figure S1. Participant recruitment. DCE-MRI, dynamic contrast-enhanced MRI; E/M-S, early/mid-secretory; HMB, heavy menstrual bleeding (≥80 ml/cycle); MBL, menstrual blood loss; MRI, magnetic resonance imaging; NMB, normal menstrual bleeding (<80 ml/cycle); P, proliferative.
