# Supplementary material for: Markers of human endometrial hypoxia can be detected in vivo and ex vivo during physiological menstruation
Source: Hum Reprod. 2021 Jan 26;36(4):941–50. doi: 10.1093/humrep/deaa379 (PMC7970728; doi:10.1093/humrep/deaa379)
Supplement: deaa379_Supplementary_TableSI [file deaa379_supplementary_tablesi.pdf]

Human Reproduction, pp. 1–1, 2021
doi:10.1093/humrep/deaa379

human reproduction

SUPPLEMENTARY DATA

Supplementary Table SI **Participant characteristics.**

Participant characteristics	Mean (range)
**Age (years)**	39 (21–47)
**Parity**	0.9 (0–4)
**BMI (kg/m^2)**	26.1 (20–39)

Stage of cycle	No. of samples	Oestradiol (pmol/l), mean (range)	Progesterone (nmol/l), mean (range)
**Menstrual**	14	158 (20–548)	1.1 (0.2–3.4)
**Proliferative**	1	348	0.5
**Early/mid-secretory**	13	400 (249–615)	39 (18.4–57.3)
