# Supplementary material for: Markers of human endometrial hypoxia can be detected in vivo and ex vivo during physiological menstruation
Source: Hum Reprod. 2021 Jan 26;36(4):941–50. doi: 10.1093/humrep/deaa379 (PMC7970728; doi:10.1093/humrep/deaa379)
Supplement: deaa379_Supplementary_TableS2 [file deaa379_supplementary_tables2.pdf]

Human Reproduction, pp. 1–1, 2021
doi:10.1093/humrep/deaa379

human reproduction

SUPPLEMENTARY DATA

Supplementary Table SII **Sequence parameters for T2*quantification and DCE-MRI.**

**Sequence**	**T2* measurement Multiecho gradient echo**	**T_1 measurement IR-TrueFISP**	**3D dynamic FLASH**
**Orientation**	Single slice through endometrium	Sagittal	Sagittal
**Voxel size**	2 × 2 × 5 mm	2 × 2 × 5 mm	2 × 2 × 5 mm
**Matrix**	192 × 192	192 × 192 × 30	192 × 192 × 30
**α/TR/TE(ms)**	18°/350/2–19	33°/5000/1.34	13°/1.96/0.74
**Other**	10 echoes, 3 averages	GRAPPA 2 AP<br>TI: 220, 300, 600, 1000, 1500, 2500 ms	GRAPPA 3 AP
**Acquisition time**	22 s	1 min 25 s per TI	2.4 s × 150 frames

FLASH, fast low angle shot; IR-TrueFISP, inversion recovery true fast imaging with steady-state precession.
