# Supplementary material for: Markers of human endometrial hypoxia can be detected in vivo and ex vivo during physiological menstruation
Source: Hum Reprod. 2021 Jan 26;36(4):941–50. doi: 10.1093/humrep/deaa379 (PMC7970728; doi:10.1093/humrep/deaa379)
Supplement: deaa379_Supplementary_TableS3 [file deaa379_supplementary_tables3.pdf]

Human Reproduction, pp. 1–1, 2021
doi:10.1093/humrep/deaa379

human reproduction

SUPPLEMENTARY DATA

Supplementary Table SIII **PCR primer and probe details.**

Gene of interest	Forward primer	Reverse primer	Roche probe
**ADM**	gcctgcccagacccttat	gtagcgcttgactcggatg	57
**VEGF-A**	cagcacaacaaatgtgaatgc	ggttcccgaaaccctgag	12
**CXCR4**	ctgtgagcagagggtccag	atgaatgtccacctcgcttt	55
**ATPB5**	agaggtcccatcaaaaccaa	tcctgctcaacactcatttcc	50
**SDHA**	tccactacatgacggagcag	ccatcttcagttctgctaaacg	70
